# Conditional survival in glioblastoma: The evolution of prognostic factors over time

**DOI:** 10.1002/ijc.70285

**Published:** 2025-12-30

**Authors:** Timothy Mueller, Flavio Vasella, Julia Velz, Stefanos Voglis, Kevin Akeret, Luis Padevit, Morton Schubert, Jonathan Weller, Sarah Brüningk, Elisabeth Rushing, Johannes Sarnthein, Dorothee Gramatzki, Levin Häni, Andreas Raabe, Anna M. Zeitlberger, Oliver Bozinov, Emilie Le Rhun, Michael Weller, Luca Regli, Marian C. Neidert

**Affiliations:** ^1^ Department of Neurosurgery, Clinical Neuroscience Center University Hospital and University of Zurich Zurich Switzerland; ^2^ Department of Neurosurgery, Inselspital Bern University Hospital and University of Bern Bern Switzerland; ^3^ Department of Neurology, Clinical Neuroscience Center University Hospital and University of Zurich Zurich Switzerland; ^4^ Department of Pediatric Neurosurgery, Kinderspital Zürich Children's University Hospital Zurich and University of Zurich Zurich Switzerland; ^5^ Department of Neurosurgery LMU University Hospital München, LMU Munich Germany; ^6^ Department of Radiation Oncology, Inselspital Bern University Hospital and University of Bern Bern Switzerland; ^7^ Department of Digital Medicine University of Bern Bern Switzerland; ^8^ Department of Neuropathology University Hospital of Zurich Zurich Switzerland; ^9^ Department of Neurosurgery, Cantonal Hospital St.Gallen HOCH Health Ostschweiz St. Gallen Switzerland

**Keywords:** high‐grade glioma, landmark analysis, long‐term survival, tumor volume

## Abstract

Conditional survival provides insights into the evolution of prognosis over time and reveals changing associations of prognostic factors during disease progression. Data on the temporal evolution of prognostic factors in glioblastoma remain scarce. We analyzed 315 patients with IDH‐wildtype glioblastoma from a prospectively collected registry (01/2008–06/2017). Our primary outcome was 12‐month conditional survival (CS), defined as the probability of surviving the next 12 months given survival for “*s*” months. This analysis was conducted at five landmarks (*s* = 0, 6, 12, 18, 24) for baseline prognostic factors, including tumor volume compartments. 12‐month conditional survival estimates at *s* = 0, 6, 12, 18, and 24 months from diagnosis were 0.51 (95% CI 0.45–0.56), 0.46 (95% CI 0.39–0.52), 0.41 (95% CI 0.33–0.49), 0.43 (95% CI 0.33–0.52), and 0.56 (95% CI 0.42–0.67), respectively. At diagnosis (*s* = 0), 12‐month survival estimates varied significantly with age >60 at diagnosis, preoperative tumor rim volume >20 cm^3^, absence of O^6^‐methylguanine‐DNA methyltransferase (MGMT) promoter methylation, postoperative KPS ≥70, residual postoperative tumor >1 cm^3^, or biopsy only. Residual tumor volume mainly influences survival in the initial months following surgery, while MGMT promoter methylation and age remain significant predictors beyond this period. These findings may refine stratification strategies in recurrent glioblastoma trials.

## What's New?

Neurooncologists rely on glioblastoma risk stratification systems for incorporating known disease‐ and patient‐specific factors at diagnosis. Although useful for general comparison and disease monitoring, these estimates are less informative for patients who survive beyond various time intervals post‐diagnosis. Here, the authors analyzed data on isocitrate dehydrogenase (*IDH*)‐wildtype glioblastoma patients to estimate conditional survival from 6 to 24 months following diagnosis. Age, *MGMT* methylation status, postoperative Karnofsky performance status, and postoperative enhancing tumor volume were identified as baseline prognostic markers. In the months after surgery, residual tumor volume dominated prognosis, whereas age at diagnosis and favorable *MGMT* status determined prognosis thereafter.

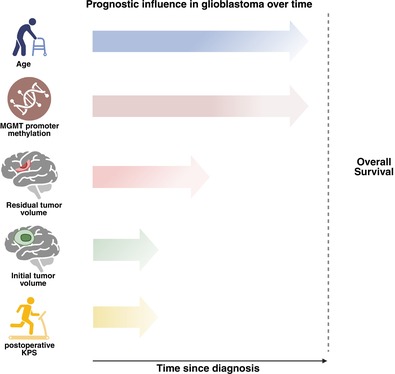

**Abbreviations**
CS: conditional survival
IDH: isocitrate dehydrogenase
KPS: Karnofsky performance status
MGMT: O^6^‐methylguanine‐DNA methyltransferase
OS: overall survival
PCR: polymerase chain reaction
TMZ: temozolomide

## 1 INTRODUCTION

Most glioblastoma risk stratification systems incorporate known disease‐ and patient‐specific risk factors at the time of diagnosis.^1^, ^2^ Although these survival estimates are important for predicting survival for newly diagnosed patients, it remains unclear whether the same risk factors retain their prognostic association throughout the disease course. Conditional survival (CS), defined as the probability that a person will survive an additional number of months after having survived “*s*” months, provides a dynamic and updated prediction of survival over the disease course which can guide treatment strategies and prognostication.^3^ CS estimates have been reported lately for a wide range of tumor types and stages.^4^, ^5^, ^6^ Some authors have studied the conditional probabilities of survival in patients with brain tumors. These reports, however, included patients diagnosed decades ago—the vast majority of patients far prior to the era of temozolomide (TMZ) and molecular markers.^7^, ^8^, ^9^ For investigating CS, it is necessary to ensure long‐term follow‐up as well as constant treatment modalities over time.^3^ Over the last decade, the definition of glioblastomas has been sharpened by the exclusion of mutations in the isocitrate dehydrogenase (*IDH1* or *IDH2*) and *histone H3* genes.^10^ O^6^‐methylguanine‐DNA methyltransferase (*MGMT*) promoter methylation has been established as a predictive marker for benefit from alkylating chemotherapy.^11^ Although there are no definitive prospective studies, several retrospective publications indicate that safe surgical resection with minimal or no residual tumor volume is associated with longer overall survival.^1^, ^12^, ^13^ The debate continues over whether this association is linked solely to contrast‐enhancing disease or also includes T2/Flair‐hyperintense residual tumor.^1^ The relationship between preoperative tumor volume and survival is less clear. This study aimed to estimate CS at 6–24 months from diagnosis in a large cohort of patients with IDH‐wildtype glioblastoma treated with standard microsurgical resection^14^ or biopsy^15^ followed by chemoradiotherapy.^16^

## 2 METHODS

### 2.1 Patient selection and clinical status

**FIGURE 1:**
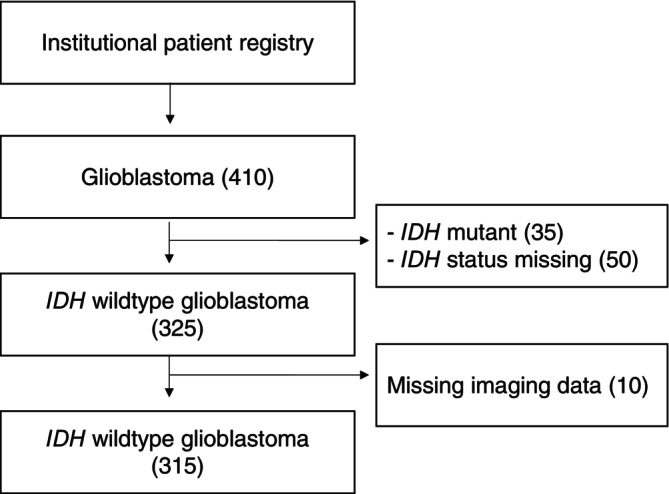
Flowchart of patient inclusion.

We included all consecutive patients that were diagnosed with glioblastoma, IDH wildtype, CNS WHO grade 4, in accordance with the WHO 2021 definition of glioblastoma,^10^ who were treated at our Neurosurgery Department at the University Hospital Zurich. Clinical information was extracted from the department's prospective patient registry.^17^ Patients with unavailable preoperative MRI studies or unavailable postoperative MRI imaging within 72 h were excluded. For biopsy patients, only the preoperative MRI scan was required for inclusion (Figure 1). Preoperative Karnofsky performance status (KPS) score was defined as the baseline general status of the patient before admission and symptom onset.

### 2.2 Molecular characterization and tumor location

As part of routine clinical diagnostics, the presence of an IDH mutation was evaluated with either immunohistochemistry or mutation‐specific polymerase chain reaction (PCR). Immunohistochemistry with a specific monoclonal antibody for the IDH1 R132H mutation was performed as described by Capper et al.^18^ Hotspots—R132 (arginine‐132) for IDH1 and R172 (arginine‐172) for IDH2—were evaluated using IDH1/Exon4 and IDH2/Exon4‐specific primers and PCR. If no mutation was detected in the examined gene regions, the tumor was classified as IDH wildtype. MGMT promoter methylation was determined by methylation‐specific PCR.^19^

### 2.3 Tumor volumetric analysis

**FIGURE 2:**
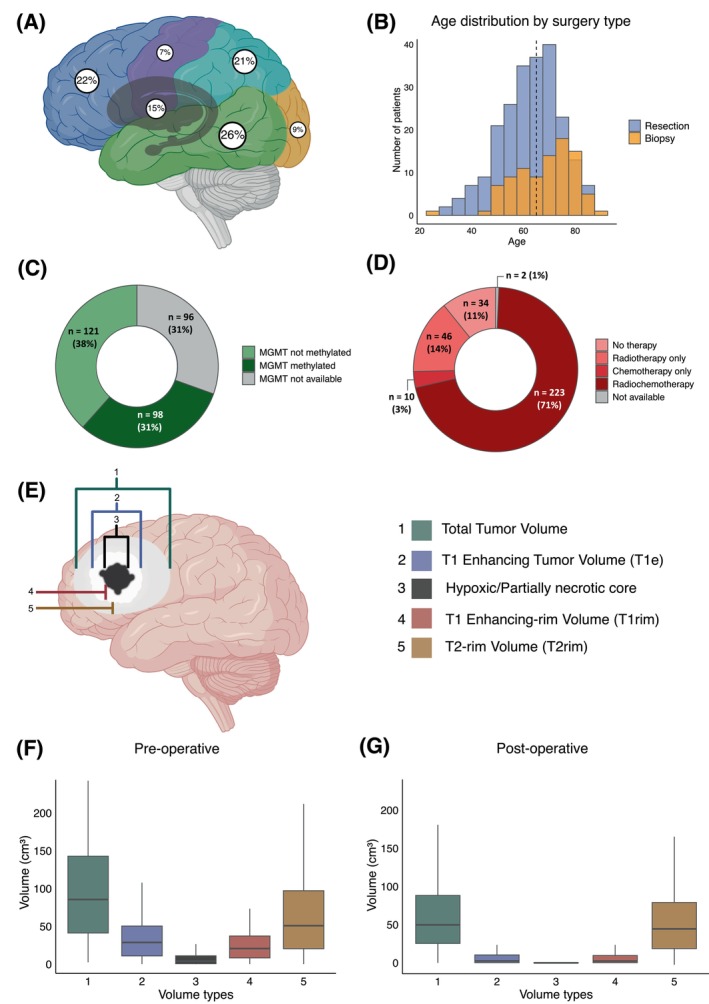
Baseline patient and tumor markers. (A) Distribution of tumor locations. (B) Histogram showing age and surgery type distribution within the cohort (dotted line = median age of 64 years) (C) *MGMT* status. (D) Distribution of therapies beyond surgery. (E) Schematic illustration of different tumor compartments and resulting tumor volumes investigated in this study. (F) Distribution of preoperative tumor volumes as defined in (E). (G) Distribution of preoperative tumor volumes as defined in (E).

Pre‐ and postoperative tumor volumes were determined in each case using T1 enhancing (T1e) and T2/FLAIR image series. Based on the T1e and FLAIR/T2 volumes, tumor volumes were grouped into 5 categories: (1) total tumor volume (including hypoxic/necrotic core, T1e and FLAIR/T2 volumes), (2) T1e tumor volume (including hypoxic/necrotic core), (3) partially necrotic/hypoxic volume (hypointense on T1), (4) T1 enhancing rim volume (T1e rim), (5) FLAIR/T2 rim volume (Figure 2). Volumetric analysis of tumors was performed using iPlan Net® (Brainlab AG, Munich, Germany). Contrast‐enhancing and FLAIR/T2 rim volumes (volumes 4 and 5) were calculated by subtracting the more internal tumor volumes.

### 2.4 Statistical analysis

All data processing and analysis steps were performed with R Studio (Version 4.3.3, R Studio Inc.) using open‐source libraries. In the initial phase of our analysis, we studied clinical, molecular, and volumetric factors at baseline that were associated with overall survival using the log‐rank test. For both pre‐ and postoperative tumor volumes, cut‐off values were determined through stepwise univariate analysis. Kaplan–Meier survival curves were generated to visualize OS for the entire cohort as well as across subgroups defined by the identified prognostic markers at baseline. We then calculated CS, defined as the probability of surviving an additional *t* months given survival of *s* months, denoted as CS (t|s). Specifically, we plotted the 12‐month CS, CS (12|s), representing the probability of surviving 12 months after having already survived s months (where *s* = 6, 12, 18, 24). This was performed for the entire cohort and for subgroups stratified by significant prognostic markers at baseline. The differences in 12‐month CS estimates between these subgroups were tested using the *z*‐test at each specific CS time point s.

Furthermore, univariate significant baseline prognostic factors were tested using a multivariate Cox proportional hazards model. In a multivariate landmark analysis^20^ these factors were also tested using a Cox proportional hazards model at 6‐, 12‐, 18‐, and 24‐months after surgery. Statistical significance was defined as *p* <.05, with Bonferroni multiple testing correction applied in the univariate setting.

## 3 RESULTS

### 3.1 Baseline characteristics

We included 315 patients diagnosed with glioblastoma, IDH wildtype between January 2008 and June 2017; 50 histologically defined glioblastomas were excluded because of missing IDH mutation status (Figure 1). The median age was 64 (range: 23–90) years, and 103 patients (33%) were females. The median OS from diagnosis for the entire cohort was 12 months (95% CI 11–13) (Table 1). Ninety‐one patients (29%) died within 6 months from diagnosis; 154 (49%), 93 (30%), and 56 (18%) patients were alive at 12, 18, and 24 months from diagnosis, respectively.

**TABLE 1:** Clinical and tumor specific characteristics of the study cohort.

Characteristics *n* = 315^a^		
Sex		
Female	103 (33%)	
Male	212 (67%)	
Age^b^	64 (23–90)	
Tumor side		
Left	123 (39%)	
Right	140 (44%)	
Both	52 (17%)	
Tumor location		
Temporal	81 (26%)	
Frontal	70 (22%)	
Parietal	67 (21%)	
Central gray^c^/limbic/insular	47 (15%)	
Occipital	28 (9%)	
Central (perirolandic)	22 (7%)	
Molecular status		
MGMT unmethylated	121 (38%)	
MGMT methylated	98 (31%)	
MGMT unknown	96 (30%)	
Therapy beyond surgery^d^		
No therapy	34 (11%)	
Chemoradiotherapy and chemotherapy maintenance (standard of care)	223 (71%)	
Radiotherapy only	46 (14%)	
Chemotherapy only	10 (3%)	
Not available	2 (1%)	

Clinical status	Preoperative	Postoperative
Seizure	77 (24%)	5 (1.6%)
Headache	109 (35%)	7 (2.2%)
Language deficit	60 (20%)	51 (17%)
Cognitive deficit	172 (55%)	126 (40%)
Motor deficit	102 (35%)	79 (27%)
Sensibility deficit	29 (9.9%)	21 (7.2%)
Visual field deficit	48 (16%)	55 (19%)
KPS^e^	100 (30–100)	80 (0–100)
NIHSS^f^	2 (0–10)	2 (0–17)
Tumor volume^g^		
Total tumor volume	94 (3–243)	55 (0–181)
T1 enhancing tumor volume	34 (0–138)	3.1 (0.0–39.5)
Hypoxic/partially necrotic core	8 (0–46)	0 (0–33)
T1 enhancing‐rim volume	26 (0–133)	2.9 (0.0–36.4)
T2‐rim volume	61 (0–216)	52 (0–180)

^a^ *n* (%); Mean (range).

^b^ Mean in years (range).

^c^ Includes: thalamus and basal ganglia.

^d^ Chemotherapy refers to treatment with Temozolomide (TMZ).

^e^ National Institute of Health Stroke Scale.

^f^ Karnofsky performance status.

^g^ Mean in cm^3^ (range).

### 3.2 Conditional survival

**FIGURE 3:**
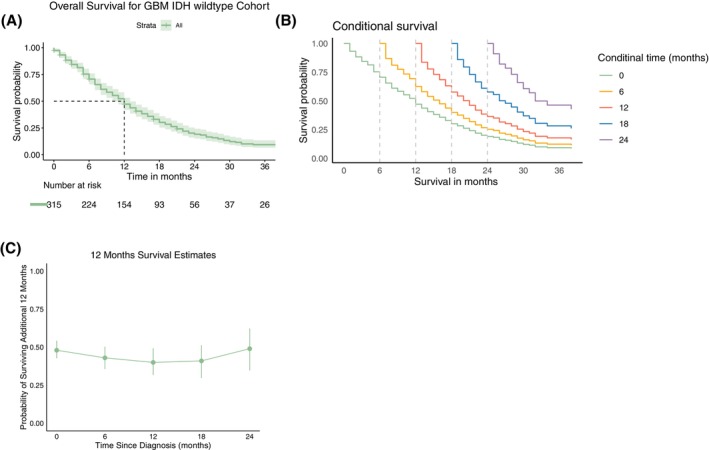
Kaplan–Meier estimator of OS and conditional survival analysis of the entire cohort (*n* = 315). (A) Kaplan–Meier estimate of overall survival (green line) including the 95% CI intervals (lighter green area) and median OS (dashed black line). (B) Conditional survival curves at 0–24 months from diagnosis. The lower curve (green) coincides with the OS curve in panel (A), whereas the upper curve (purple) for instance provides the survival under the condition that a patient has already survived 24 months. Conditional time points are 6, 12, 18 and 24 (dashed black). (C) 12‐month conditional survival estimates (and 95% CI) at diagnosis and at 6–24 months since diagnosis.

Median CS estimates for *s* = 0, 6, 12, 18, and 24 months from diagnosis were 12 (95% CI 11–13), 16 (95% CI 14–18), 21 (95% CI 19–23), 27 (95% CI 24–30), and 33 (95% CI: 30–42) (Figure 3A/B). Twelve‐month CS estimates at *s* = 0, 6, 12, 18, and 24 months from diagnosis were 0.51 (95% CI 0.45–0.56), 0.46 (95% CI 0.39–0.52), 0.41 (95% CI 0.33–0.49), 0.43 (95% CI 0.33–0.52), and 0.56 (95% CI 0.42–0.67), respectively. The conditional probability of surviving an additional year after reaching 24 months post‐diagnosis thus exceeded the 12‐month survival rate at baseline (Figure 3C).

### 3.3 Univariate analysis of prognostic factors at baseline

On univariate analysis, age ≤60 (HR = 0.48 [95% CI = 0.37–0.61], *p* <.001), surgery type (resection versus biopsy) (HR = 0.44 [95% CI = 0.34–0.57], *p* <.001), postoperative residual T1 enhancing tumor volume (<1 cm^3^) (HR = 0.53 [95% CI = 0.42–0.68], *p* = <.001), postoperative KPS ≥70 (HR = 0.39 [95% CI = 0.27–0.56], *p* = .001), methylated *MGMT* promoter (HR = 0.57 [95% CI = 0.43–0.76], *p* = <.001), treatment beyond surgery (chemo and/or radiotherapy) (HR = 0.09 [95% CI = 0.06–0.14], *p* <.001) and preoperative T1 enhancing‐rim tumor volume (<20 cm^3^) (HR = 0.77 [95% CI = 0.61–0.98], *p* = 0.032), were associated with increased survival at baseline. Sex, preoperative total, T1 enhancing, T2‐rim and partially necrotic core tumor volumes along with postoperative T2 tumor volume were not associated with OS (Supplementary Tables S1 and S2).

### 3.4 Baseline prognostic factors over time (univariate)

**FIGURE 4:**
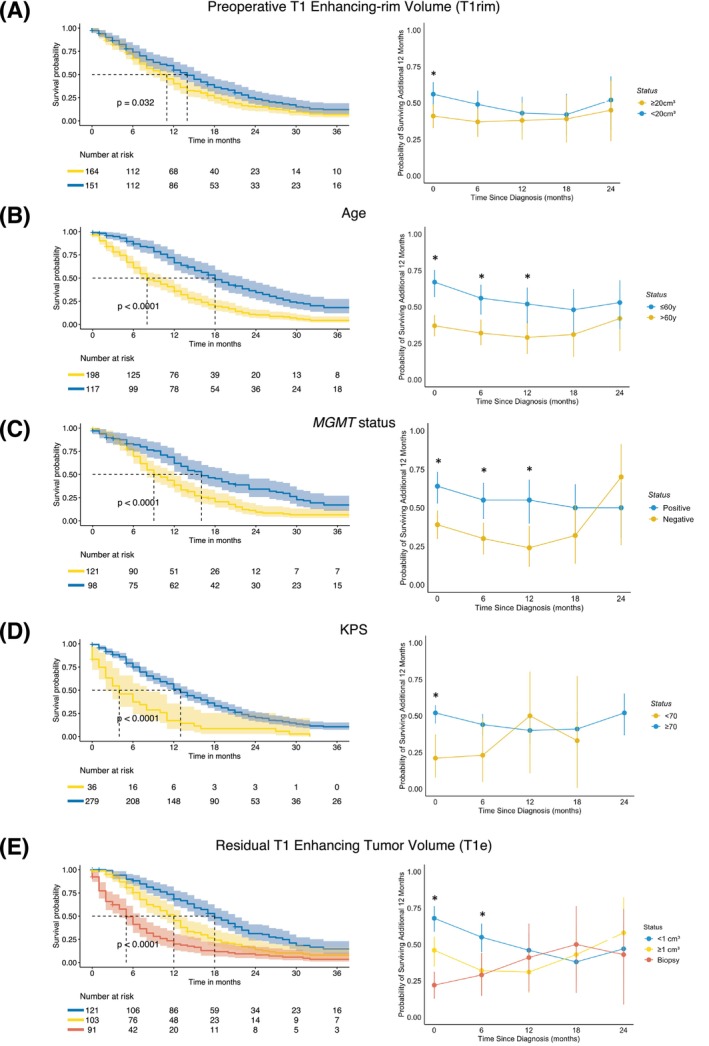
Kaplan–Meier estimates of OS (left side, including 95% CI) and 12‐month CS probability estimates (right side, including 95% CI) from each time point s (= 0, 6, 12, 18, 24 months) stratified by (A) Preoperative T1 enhancing‐rim tumor volume, (B) Age, (C) MGMT Promoter methylation status, (D) postoperative KPS score, and (E) post‐operative residual T1 enhancing tumor volume. *Significant difference (*p* <.05).

The Kaplan–Meier estimator of OS and the 12‐month CS probability estimates at each time point s (= 6, 12, 18, 24 months) stratified by preoperative T1e tumor volume, age, *MGMT* promoter methylation status, postoperative KPS, and postoperative residual T1e tumor volume are shown in Figure 4. After the first few months after diagnosis, the initial survival advantage associated with smaller preoperative T1‐enhancing rim volumes (<20 cm^3^) diminished, and survival rates equalized with those of patients who had larger initial tumor volumes (>20 cm^3^) (Figure 4A). Patients who are ≤60 years at baseline had a rather constant 12‐month CS of about 0.60, whereas patients older than 60 years had lower 12‐month CS of about 0.30–0.4, with a decreasing gap between both groups over time (Figure 4B). Methylated *MGMT* promoter status was associated with an increased survival at baseline, with only a small decrement over time compared to unmethylated *MGMT* promoter tumors until the 12 months landmark and lost significance thereafter (Figure 4C). The initial association of a favorable survival outcome and a favorable performance score (KPS) no longer persisted beyond the 0 months landmark (Figure 4D). Gross total resection with little T1 enhancing residual tumor (<1 cm^3^) was associated with a longer OS at baseline with an effect magnitude that steadily decreased with additional months survived, also when compared to residual tumor ≥1 cm^3^ or biopsy only (Figure 4E).

### 3.5 Baseline prognostic factors over time (multivariate)

**FIGURE 5:**
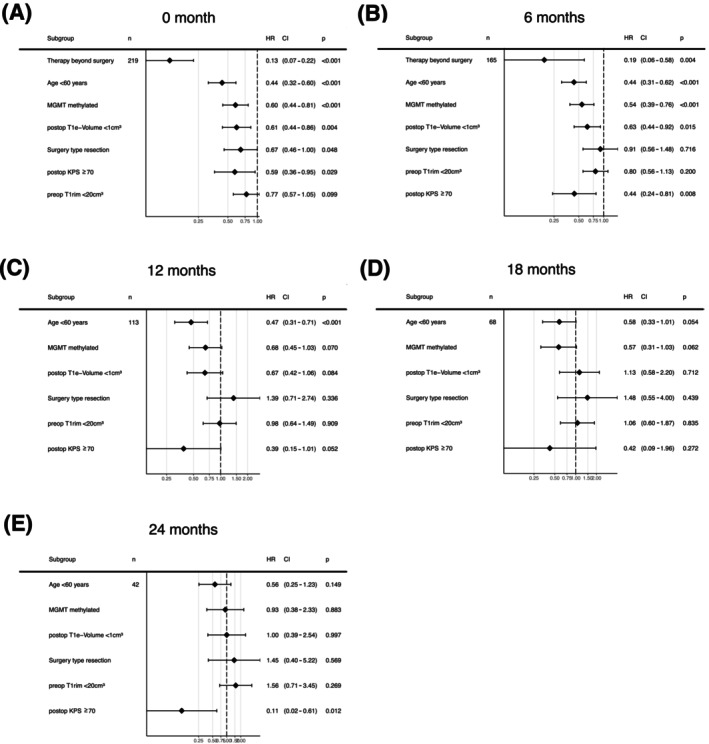
Multivariate analysis using Cox proportional hazard model estimating the hazard ratio for survival of baseline prognostic factors. (A) At baseline. (B) 6 months post‐surgery. (C) 12 months post‐surgery 12. (D) 18 months post‐surgery. (E) 24 months post‐surgery.

On multivariate analysis, age <60, surgery type (resection), preoperative T1 enhancing‐rim tumor volume (<20 cm^3^), *MGMT* promoter methylation, KPS >70 along with postoperative residual T1 enhancing tumor volume (<1 cm^3^) were associated with decreased OS at baseline (Figure 5A). In the multivariate landmark analysis at time points 6, 12, 18, and 24 months (Figure 5B–E), only age at diagnosis and *MGMT* promoter methylation approached significance at 18 months.

## 4 DISCUSSION

Our study provides a novel look at the analysis of clinical, surgical, and molecular factors at different time points, thereby illustrating the temporal evolution of prognostic factors in patients with glioblastoma. While selected variables assessed in the present study align with well‐established prognostic determinants at baseline,^1^ changes in the prognostic importance of different compartments of pre‐ and postoperative tumor volumes and molecular markers have not been explored in detail over time. Estimates of subsequent survival probabilities after a patient has survived for a certain number of months cannot be extrapolated from the standard Kaplan–Meier curve at baseline.

Consistent with previous studies,^2^, ^13^, ^14^, ^21^ we identified age, *MGMT* promoter methylation status, postoperative KPS, and postoperative enhancing tumor volume as baseline prognostic markers. Our detailed tumor compartment analysis revealed that preoperative T1‐enhancing rim volume serves as a baseline prognostic marker, potentially serving as a surrogate for vital tumor burden, also reflecting surrounding tumor infiltration. In contrast, neither the preoperative T2/FLAIR tumor volume, representing a mixture of vasogenic edema and tumor cell infiltration, nor the postoperative T2/FLAIR volume was associated with prognosis.

Previous reports on CS for glioblastoma patients involve tumors diagnosed over two decades ago,^7^, ^8^, ^9^ prior to molecular characterization (such as IDH mutation/*MGMT* promoter methylation) and current standard treatment regimens. In a histologically defined cohort of 498 glioblastoma patients, Polley et al.^9^ demonstrated that the 12‐month CS rate after surviving 1 year post‐diagnosis was 0.35 (95% CI: 0.29–0.40), which is comparable to our estimate of 0.41 (95% CI: 0.33–0.49). In a more recent report from the EORTC 1419 ETERNITY study, Hertler et al.^22^ found that *MGMT* promoter methylation, younger age, and gross total resection were significantly more common in patients who survived beyond 5 years compared to the general glioblastoma IDH wildtype population. However, their Cox regression analysis showed that none of these factors remained prognostic among long‐term survivors, suggesting that these prognostic factors have already exerted their association with survival after 5 years. With our 12‐month CS analysis, we demonstrate that predefined factors, such as patient age at diagnosis and the currently non‐modifiable MGMT promoter methylation status,^23^ maintain a consistent association with survival from diagnosis up to 18 months. Alternatively, the association of resection with minimal post‐operative tumor residual diminishes after the first 6 months. These findings are consistent with our multivariate landmark analysis, in which patient age <60 and MGMT promoter methylation approached significance for 18‐month survival.

Postoperative KPS <70 lost prognostic significance within the first 6 months, likely reflecting its role as a surrogate for preexisting severe and persistent impairment. Long‐term deterioration of clinical status, however, remains an important prognostic factor and may more reliably guide subsequent clinical decision‐making than a CS model based solely on postoperative KPS. A structured classification system, such as the Therapy‐Disability‐Neurology (TDN)^24^ could further enhance the assessment of baseline complications and help differentiate survival trajectories over time.

Several important limitations of this study should be acknowledged. The single‐center design and relatively small sample size limit the statistical power, particularly as the number of patients decreases at successive time points. This highlights an inherent challenge of CS analyses in highly aggressive malignancies such as glioblastoma, where the at‐risk population declines rapidly over time. Consequently, estimates derived at later intervals are based on progressively smaller subgroups and should be interpreted with caution.

Furthermore, our analysis was restricted to a limited number of clinical and molecular markers. Other alterations with known prognostic impact^25^, ^26^ (e.g., EGFR, PTEN, NF1, PDGFRA, TP53) were not included. Similarly, prognostic factors relevant in the later course of disease—such as tumor location, feasibility of local treatment at recurrence (re‐resection^27^ or re‐irradiation^28^), and salvage therapies like CCNU or bevacizumab,^29^, ^30^ were not systematically addressed. These increasingly complex and individualized treatment trajectories of long‐term glioblastoma survivors are not captured in our design, which limits the direct clinical applicability of our findings.

Future studies with larger, multicenter cohorts, broader molecular profiling, and more detailed treatment documentation are warranted to refine CS models and strengthen their value for patient management and counseling.

We conclude that the association of genetic, surgery‐related, and clinicopathologic factors with OS changes over time for glioblastoma patients. Specifically, residual tumor volume dominates prognosis in the months after surgery, whereas age at diagnosis and favorable *MGMT* status determine the prognosis thereafter. Findings may refine stratification strategies in recurrent glioblastoma trials.

## AUTHOR CONTRIBUTIONS

**Timothy Mueller:** Conceptualization; methodology; formal analysis; data curation; writing – review and editing; writing – original draft; visualization. **Flavio Vasella:** Writing – review and editing; data curation. **Julia Velz:** Data curation; writing – review and editing. **Stefanos Voglis:** Data curation; writing – review and editing. **Kevin Akeret:** Data curation; writing – review and editing. **Luis Padevit:** Writing – review and editing; data curation. **Jonathan Weller:** Data curation; software. **Sarah Brüningk:** Validation; formal analysis. **Elisabeth Rushing:** Data curation. **Johannes Sarnthein:** Project administration; supervision. **Dorothee Gramatzki:** Writing – review and editing; data curation. **Levin Häni:** Writing – review and editing; conceptualization. **Andreas Raabe:** Writing – review and editing. **Anna M. Zeitlberger:** Writing – review and editing. **Oliver Bozinov:** Writing – review and editing; resources. **Emilie Le Rhun:** Writing – review and editing; conceptualization. **Michael Weller:** Conceptualization; writing – review and editing; resources. **Luca Regli:** Conceptualization; resources; writing – review and editing. **Marian C. Neidert:** Conceptualization; investigation; writing – review and editing; formal analysis; supervision; resources; writing – original draft; visualization; methodology. **Morton Schubert:** Data curation.

## CONFLICT OF INTEREST STATEMENT

Prof. M. Weller reports receiving research grants from Versameb, Novartis, and Quercis. He has received honoraria for lectures and/or participation in advisory boards from Nuvation, Bayer, CureVac, Hemerion, Iqvia, Medac, Novartis, Orbus, Pfizer, Philogen, Roche, and Servier. Dr. E. le Rhun reports receiving research grants from BMS and Servier. She has received honoraria for lectures, participation in advisory boards, and consulting from AstraZeneca, Daiichi Sankyo, Bayer, Biodexa/ Sitoxi, Janssen, Leo Pharma, Medac, myTomorrows, Pfizer, Pierre Fabre, Roche, Seattle Genetics, and Servier. Prof. J. Sarnthein reports receiving honoraria for consulting or lectures from inomed. Prof. M.C. Neidert reports receiving a research grant from Novocure and honoraria for consulting or lectures from WISE, MSD, Osteopore, Servier, and Terumo. All other authors declare that they have no conflicts of interest.

## ETHICS STATEMENT

The study was approved by the local ethical review board (“Kantonale Ethikkommission Zürich”, identifier PB‐2017‐00093), registered at clinicaltrials.gov (NCT01628406). Individual patient consent was waived.

## Supporting information


**Supplementary Table S1.** Univariate analysis of pre‐ and postoperave tumor.
**Supplementary Table S2.** Univariate analysis of baseline paent characteriscs.

## Data Availability

The data supporting the findings of this study are available from the corresponding author upon reasonable request. All source code is publicly available on GitHub (https://github.com/muellertimothy/Conditional-survival-in-GBM).
