# Supplementary material for: Conditional survival in glioblastoma: The evolution of prognostic factors over time
Source: Int J Cancer. 2025 Dec 30;158(10):2571–80. doi: 10.1002/ijc.70285 (PMC12996738; doi:10.1002/ijc.70285)
Supplement: Supplementary file 1 — Supplementary Table S1. Univariate analysis of pre‐ and postoperave tumor. Supplementary Table S2. Univariate analysis of baseline paent characteriscs. [file IJC-158-2571-s001.pdf]

Conditional survival in glioblastoma: The evolution of prognostic factors over time

Timothy Mueller, Flavio Vasella, Julia Velz, Stefanos Voglis, Kevin Akeret, Luis Padevit, Morton Schubert, Jonathan Weller, Sarah Brüningk, Elisabeth Rushing, Johannes Sarnthein, Dorothee Gramatzki, Levin Häni, Andreas Raabe, Anna M. Zeitlberger, Oliver Bozinov, Emilie Le Rhun, Michael Weller, Luca Regli, and Marian C. Neidert

Table of Contents (Supplementary Material)

Supplementary Table S1. Univariate analysis of pre- and postoperative tumor volume compartments
Supplementary Table S2. Univariate analysis of baseline patient characteristics

Supplementary Table S1 Univariate pre- and postoperative tumor volume compartments analysis

Volume Type	Volume (cm³)	HR	95%-CI	p-value
preoperative Total Tumor Volume	5	1.61	0.52–5.03	0.412
	10	1.18	0.70–1.99	0.532
	20	1.1	0.77–1.57	0.594
	30	1.2	0.89–1.60	0.231
	40	1.2	0.92–1.57	0.174
	50	1.24	0.96–1.59	0.098
	60	1.17	0.92–1.49	0.195
	90	1.03	0.82–1.30	0.8
preoperative T1 Enhancing Tumor Volume (T1e)	5	1.12	0.82–1.54	0.473
	10	1.15	0.88–1.51	0.307
	20	1.07	0.84–1.36	0.579
	30	1.2	0.95–1.52	0.121
	40	1.08	0.85–1.38	0.527
	50	1.14	0.88–1.49	0.33
	60	1.22	0.89–1.67	0.216
	90	1.33	0.77–2.28	0.305
preoperative Hypoxic/ necrotic Voume	5	1.02	0.81–1.29	0.867
	10	0.96	0.75–1.24	0.769
	20	0.91	0.62–1.33	0.625
	30	1.38	0.81–2.37	0.241
	40	1.44	0.54–3.87	0.47
preoperative T1 Enhancing-rim Volume (T1rim)	5	1.09	0.81–1.46	0.589
	10	1.14	0.88–1.47	0.331
	20	1.29	1.02–1.63	**0.032**
	30	1.27	0.99–1.63	0.059
	40	1.16	0.88–1.54	0.287
	50	1.07	0.75–1.53	0.701
	60	1.12	0.72–1.73	0.616
	90	0.88	0.33–2.37	0.802
preoperative T2-rim Volume (T2rim)	5	0.85	0.54–1.32	0.465
	10	1.01	0.71–1.45	0.94
	20	1.14	0.87–1.48	0.345
	30	1.13	0.89–1.44	0.326
	40	1.09	0.87–1.38	0.451
	50	1.09	0.86–1.38	0.466
	60	0.98	0.77–1.24	0.872
	90	0.89	0.69–1.16	0.403
postoperative Total Tumor Volume	0	1.07	0.15–7.62	0.948
	1	1.07	0.15–7.62	0.948
	2	1.53	0.49–4.77	0.467
	3	1.24	0.51–3.02	0.632
	4	1.15	0.59–2.24	0.681
	5	1.18	0.66–2.11	0.571
	6	1.15	0.67–1.98	0.606
	10	1.14	0.77–1.67	0.515
postoperative T1 Enhancing Tumor Volume (T1e)	0	1.73	1.27–2.36	**<0.001**
	1	1.88	1.48–2.39	**<0.001**
	2	1.72	1.36–2.18	**<0.001**
	3	1.9	1.50–2.40	**<0.001**
	4	2.06	1.62–2.62	**<0.001**
	5	1.99	1.56–2.53	**<0.001**
	6	2.13	1.66–2.73	**<0.001**
	10	1.92	1.47–2.52	**<0.001**
postoperative T2-rim Volume (T2rim)	0	1.07	0.15–7.62	0.948
	1	0.8	0.39–1.61	0.526
	2	0.69	0.38–1.23	0.208
	3	0.65	0.39–1.09	1.103
	4	0.84	0.53–1.35	0.474
	5	0.92	0.59–1.42	0.691
	6	0.89	0.59–1.35	0.599
	10	0.97	0.70–1.36	0.866

Supplementary Table S2 Univariate analysis of patient baseline factors

Characteristic	Group	HR	95%-CI	p-value
sex	male	0.93	0.73–1.20	0.587
preoperative KPS	≥90	0.88	0.55–1.41	0.595
surgery type	resection	0.44	0.34–0.57	**<0.001**
Age	≤60 years	0.48	0.37–0.61	**<0.001**
postoperative KPS	≥90	0.57	0.45–0.72	**<0.001**
